# Landscape and Saturation Analysis of Mutations Associated With Race in Cancer Genomes by Clinical Sequencing

**DOI:** 10.1093/oncolo/oyad341

**Published:** 2024-01-31

**Authors:** Maishara Muquith, David Hsiehchen

**Affiliations:** University of Texas Southwestern Medical School, Dallas, TX, USA; Division of Hematology and Oncology, Department of Internal Medicine, University of Texas Southwestern Medical Center, Dallas, TX, USA

**Keywords:** cancer genomes, disparities in cancer care, GENIE

## Abstract

Differences in cancer genomes between racial groups may impact tumor biology and health disparities. However, the discovery of race-associated mutations is constrained by the limited representation and sample size of different racial groups in prior genomic studies. We evaluated the influence of race on the frequency of gene mutations using the Genomics, Evidence, Neoplasia, Information, Exchange database, a large genomic dataset aggregated from clinical sequencing. Matched cohort analyses were used to identify histology-specific race-associated mutations including increased *TERT* promoter mutations in Black and Asian patients with gliomas and bladder cancers, and a decreased frequency of mutations in DNA repair pathway genes and subunits of the SWI/SNF chromatin complex in Asian and Black patients across multiple cancer types. The distribution of actionable mutations in oncogenes was also race-specific, demonstrating how targeted therapies may have a disparate impact on racial groups. Down-sampling analyses indicate that larger sample sizes are likely to discover more race-associated mutations. These results provide a resource to understand differences in cancer genomes between racial groups which may inform the design of clinical studies and patient recruitment strategies in biomarker trials.

## Teaser

This article evaluates the influence of race on the frequency of gene mutations using the Genomics, Evidence, Neoplasia, Information, Exchange database, a large genomic dataset aggregated from clinical sequencing.

**Implications for Practice**

Characterizing genetic differences in cancers between racial groups may enhance precision medicine by identifying biological factors that impact treatment outcomes and clarifying the genetic basis of cancer disparities to inform public health strategies, clinical guidelines, and policies to reduce inequities. Herein, we leveraged the largest cancer genome databases to evaluate the impact of race on the frequency of gene mutations in cancers. We identified numerous genes more frequently mutated in a racial group across many cancer histologies and distinct distributions of hotspot mutations in common oncogenes. In addition, down-sampling analyses showed that larger sample sizes are likely to discover more race-associated mutations. Our analysis provides a resource to better understand the genetic contribution to cancer disparities with implications for drug discovery efforts and understanding the relevance of precision oncology to all patients.

## Introduction

The incidence and outcomes of multiple cancer types are robustly associated with race.^1-5^ This has been linked to socioeconomic barriers, behavioral factors, financial limitations, concurrent illness, access to care, treatment disparities, and other inequities within healthcare systems.^1-10^ However, disparate outcomes between races still persist even within cohorts of cancer patients in randomized trials where baseline characteristics and treatment procedures are more uniform.^11-13^ Although, other variables contributing to racial disparities in cancer disease burden likely remain to be identified, biological differences in cancers between racial groups may be important determinants of cancer incidence and outcomes after treatment. Genome profiling studies in individual cancer types have demonstrated differences in cancer genomes in various cancers including lung, breast, prostate, gastric, endometrial, and colon cancers.^14-24^ Moreover, the distribution of tumor mutation burden, an emerging prognostic and predictive molecular marker, is distinct between racial groups in several cancer types.^25-27^ The prevalence of genetic alterations with therapeutic relevance has also been shown to be different between race, suggesting that race-specific mechanisms may underly treatment resistance or actionability.^28,29^

A limitation of prior genomic studies is the underrepresentation of Asian and Black patients which is likely associated with insufficient statistical power to comprehensively detect race-associated mutations.^30-32^ To overcome this barrier, we interrogated the Genomics, Evidence, Neoplasia, Information, Exchange (GENIE) database (v9.1) which is among the largest source of genomic and clinical data including clinical sequencing of Asian and Black individuals, and is nearly a magnitude larger than The Cancer Genome Atlast (TCGA). We used GENIE to discover race-associated molecular alterations among advanced cancers and to perform down-sampling analyses to assess the utility of further sequencing efforts.

## Methods

GENIE is a cancer registry including clinical-grade genomic data obtained as a component of routine clinical practice for patients with cancer. The final GENIE cohort was comprised of 71 008 patients (self-reported gender: 57% female and 43% male) with solid cancers and race data encompassing 51 cancer types including 61 864 White, 4801 Black, and 4343 Asian individuals.

Sequencing data from GENIE are generated in Clinical Laboratory Improvement Amendments/ISO-certified and experienced molecular pathology laboratories ensuring high-quality variant calls. Version 9.1 of the genomic data and matching clinical attributes including sex, self-reported race, birth year, age at sequencing, and cancer type were downloaded from the GENIE data portal (genie.cbioportal.org/). Cases missing race data and duplicate entries were removed. Mutations were further refined to alterations predicted to be damaging by PolyPhen and deleterious according to Sorting Intolerant From Tolerant. Genes analyzed were refined to 178 cancer driver genes that were sequenced in at least 80% of all cancers (Supplementary Table S1) given differences in gene coverage among sequencing platforms. Analyses of mutation frequencies were adjusted for gene panel coverage of the molecular alteration or calculated by using the number of cases in which the gene of interest was sequenced as the denominator. Given that all sequencing platforms reported single-nucleotide variants and small delins but not copy number alterations and structural variants, we focused our analysis on the former 2 mutation types.

### Statistical Analysis

Multivariate regression models were generated for each comparison (Black vs White patients and Asian vs White patients) to assess the effects of race on the frequency of molecular alterations while controlling for possible confounders such as cancer type, age, sex, histology, and gene panel testing. Because histology data were not uniformly reported across contributing centers for all cancer types, major histologic subtypes were abstracted by manual review for the following cancer types: non–small cell lung cancer (NSCLC; adenocarcinoma, squamous), breast (lobular, ductal, and mixed), thyroid (papillary, other), endometrial (mucinous/endometriod, serous/clear cell), glioma (high-grade/glioblastoma, other), and hepatobiliary (hepatocellular, bile duct). A dummy variable was used for cases where histology data were ambiguous. For cases where genes were not included in the sequencing gene panel, alterations were classified as missing and thus excluded using complete case analyses. Results were corrected for multiple hypotheses using the Benjamini-Hochberg procedure.

To elucidate race-associated mutations specific to each cancer type, we performed separate matched cohort analysis for each comparison (Black vs White patients and Asian vs White patients). Cohorts were created using a 1:4-5 matching ratio of Black or Asian patients to White patients that were exactly matched on cancer type, major histology subtypes, sex, and age (5-year increments). This analysis was restricted to 22 cancer types with at least 25 Black and Asian individuals and a sufficient number of matching White patients. Results were considered statistically significant using an FDR threshold of 0.1.

To assess the relationship between the number of significant race-associated mutations and the sample size of the dataset, we performed down-sampling analyses for each comparison (Black vs White patients and Asian vs White patients). To conduct this, we repeated our pan-cancer analysis using multivariate regression models on subsets of patients of decreasing size at 1% decrements. Each subset was independently generated by random selection of patients so that each decrement did not necessarily exclude the same patients across subsets. The number of race-associated genes that remained significant based on a *P*-value <.05 at each smaller set size was plotted as a smoothed function of set size.

## Results

### GENIE Cohort and Statistical Power

The scale of the GENIE cohort which is markedly larger than prior clinicogenomic studies provides improved statistical power to detect race-associated mutations compared to prior analyses. This is because a larger absolute sample size of either Black, Asian, and White patients would enable the detection of smaller differences in mutation frequencies between race using the same threshold for statistical significance or to detect the same difference in mutation frequencies between race with greater statistical confidence. To support this notion, we simulated the discovery rate of differentially mutated genes between Black and White patients in both the GENIE and TCGA (*N* = 10 678 patients) cohorts over a range of odds ratio (OR) in mutation frequencies in Supplementary Fig. 1. This analysis shows that with the larger sample size of the GENIE versus the TCGA cohort, the power to detect any OR is always greater in the GENIE cohort. In addition, as the mutation frequency becomes more common, the power to detect any OR increases more rapidly in the GENIE cohort. For instance, while prior pan-cancer TCGA analyses would have 40% power to detect race-associated mutations of at least 1% prevalence and an OR of 2 in a comparison between White and Black patients, a similar analysis in GENIE would have nearly 100% power (Supplementary Fig. S1).

### Pan-Cancer Gene-Level Analysis of Race-Associated Mutations

Logistic regression analyses adjusting for cancer type and histology of major cancer types with available data, sex, and age were performed to test the association of race with gene-level mutations. In a comparison between Black and White patients using a false discovery rate of 0.1, *TP53* mutations were more frequent in Black individuals, while mutations in *BCOR*, *IDH1*, and *VHL* were less frequent (Supplementary Fig. S2). In a comparison between Asian and White patients, mutations in *NOTCH2*, *KDM6A*, *KRAS*, and *BRAF* were less frequent in Asian individuals (Supplementary Fig. S2). In contrast, *EGFR*, *BARD1*, *FOXA1*, *JAK3*, *JAK1*, and *TERT* promoter mutations were more prevalent in Asian patients (Supplementary Fig. S1).

### Matched Cohort Analysis of Gene-Level Race-Associated Mutations in Cancer Types

To elucidate race-associated mutations among specific cancer types, we used a 1:4-5 matching ratio of Black or Asian patients to White patients to create cohorts exactly matched on cancer type/histology, sex, and age (5-year increments) among 22 cancer types with sufficient sample size.

**Figure 1.**
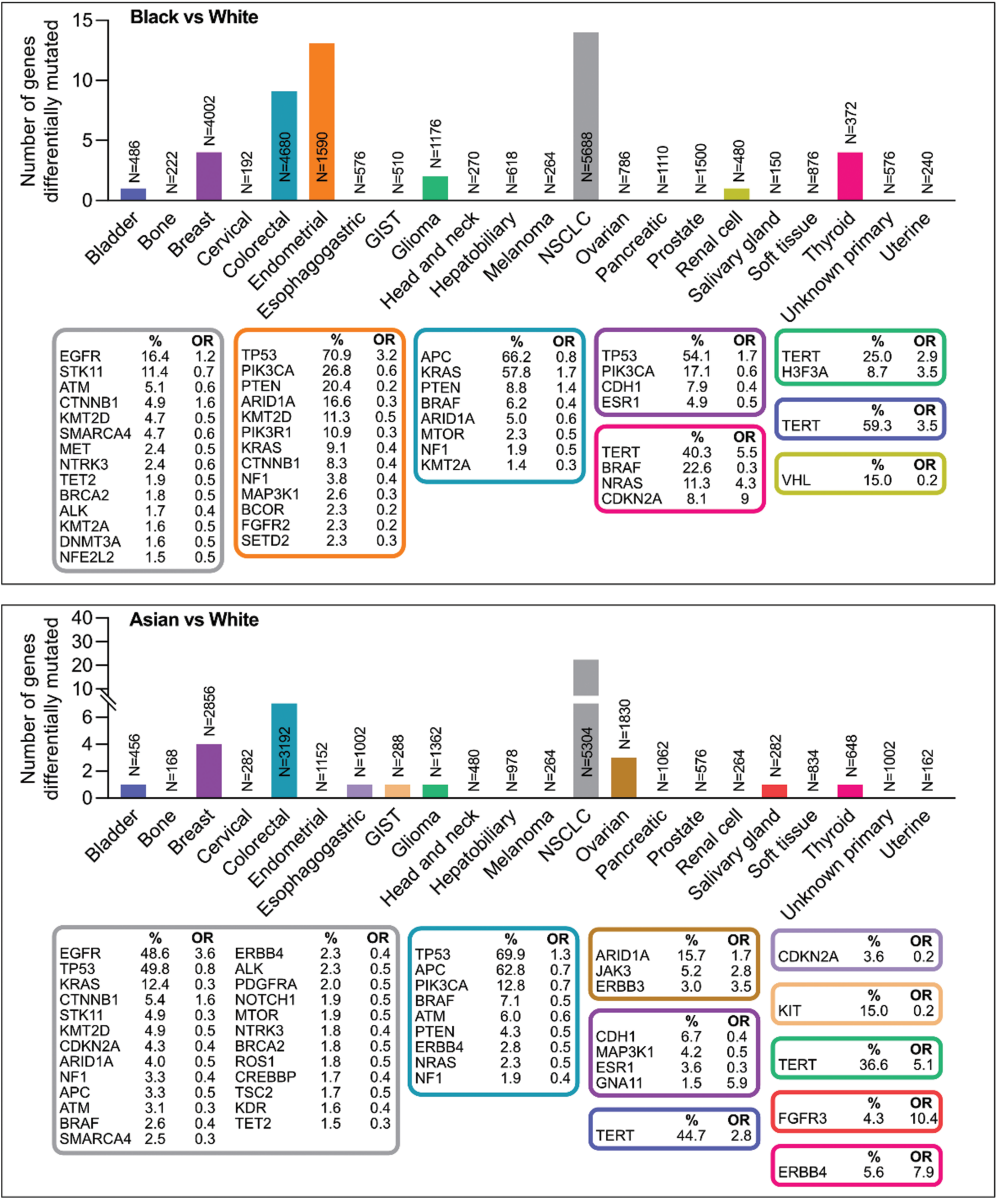
Gene-level cancer type-specific race-associated molecular alterations. Mutation frequencies between age-, sex-, and cancer type-matched cohorts (1:4-5 matching ratio of Asian or Black patients to White patients) were compared using Fisher’s exact test among 22 cancer types using an FDR threshold of 0.1 to define significance. Insets show gene-level race-associated mutations for each cancer type matched by color. Percentages indicate the prevalence of mutations in Asian or Black patients in the matched cohort. OR indicate the odds of a mutation in Asian or Black patients.

Using an FDR threshold of 0.1 and a minimal gene frequency threshold of 1% in Black and Asian patients, there were significant gene-level race-associated mutations in 8 cancer types for Black patients and 10 cancer types for Asian patients (Fig. 1). These mutations were found in overlapping cancer types including NSCLC, colorectal, bladder, breast, glioma, and thyroid cancers, while mutations exclusively associated with Black patients were observed in renal cell cancers (Fig. 1). In contrast, mutations exclusively associated with Asian patients were present in esophagogastric cancers, gastrointestinal stromal tumors, ovarian cancers, and salivary gland tumors (Fig. 1). Even among cancer types where mutations associated with Black or Asian patients were detected, the degree of overlapping genes harboring race-associated mutations across cancer types varied considerably (Fig. 1).

Cancer types harboring the greatest number of race-associated mutations included NSCLC and colorectal cancers for both Black and Asian patients (Fig. 1). Among the most prevalent mutated genes in NSCLC, *EGFR* and *CTNNB1* were more frequently altered in Black and Asian patients, with a decreased prevalence of other driver gene mutations (Fig. 1). Multiple driver genes of relatively high prevalence in other cancer types were associated with race, including an increased frequency of *KRAS* and *PTEN* mutations but decreased prevalence of *APC* and *BRAF* mutations in Black patients with colorectal cancer (Fig. 1). *TERT* promoter mutations were more frequent among Asian and Black individuals in bladder cancers and gliomas, but were only increased in Black patients with thyroid cancers. Patterns of race-associated mutation frequencies across cancer types were not necessarily concordant, with *TP53* mutations in Asian patients being less frequent in NSCLC, but more frequent in colorectal cancer (Fig. 1). In Black patients, *CTNNB1* mutations were more frequent in NSCLC, but less frequent in endometrial cancers (Fig. 1).

The scale of the GENIE cohort enabled the detection of differences in molecular alterations in less prevalent genes within each cancer type. For example, *CDKN2A* and *NRAS* alterations, which were mutated in 8.1% and 11.3% of thyroid cancers respectively in Black patients, were more common in Black than White patients (Fig. 1). Similarly, while only 8.7% of gliomas in Black patients had *H3F3A* mutation, this gene was more likely to be differentially mutated in Black than White individuals (Fig. 1). Less prevalent genes of potential therapeutic relevance were also differentially mutated between race, including a decreased frequency of mutations in DNA repair pathway genes (*ATM*, *BRCA2*, and *BRCA1*) and subunits of the SWItch/sucrose non-fermentable (SWI/SNF) chromatin complex (*SMARCA4* and *ARID1A*) in both Asian and Black patients across multiple cancer types (Fig. 1).

### Mutation-Level Analysis of Race-Associated Alterations in Oncogenes

Oncogenic mutations universally result in an activated protein, but biological effects of different mutations in the same oncogene can result in disparate clinical phenotypes and have distinct therapeutic implications.^33-36^ Using matched GENIE cohorts, we examined the distribution of hotspot alterations at the mutation-level for the most commonly altered oncogenes including *KRAS*, *PIK3CA*, *BRAF*, and *EGFR* among cancer types where the oncogene of interest was frequently mutated.

**Figure 2.**
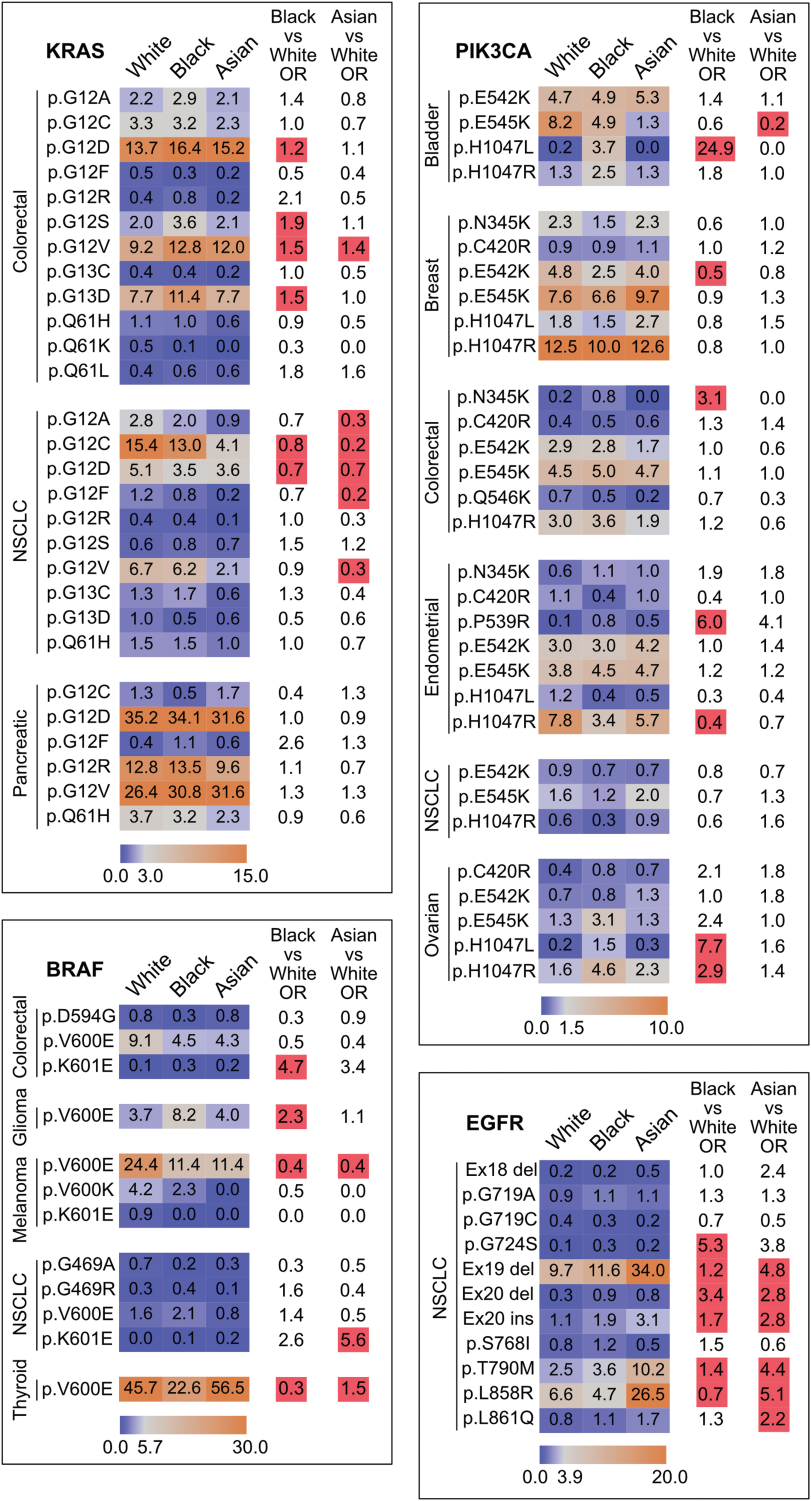
Oncogene mutations associated with race. Distribution of *KRAS*, *PIK3CA*, *BRAF*, and *EGFR* mutations among cancer types where the oncogene of interest is frequently mutated. Heatmaps depict the prevalence of oncogene mutations with orange indicating the most frequent alterations and blue indicating the least frequent alterations. Statistically significant OR are highlighted in red.

Consistent with prior findings, KRAS G12C mutations were less frequent in Asian and Black patients compared to White patients in NSCLC, but not other cancers (Fig. 2).^28^ Other hotspot KRAS mutations affecting amino acid glycine 12 including G12A, G12D, G12F, and G12V were less frequent in Asian and Black patients in NSCLC. In colorectal cancers, the prevalence of KRAS G12D, G12S, G12V, and G13D mutations was greater in Black patients (Fig. 2).

Multiple PIK3CA mutations were more frequent in Black patients, including H1047L in bladder cancer, N345K in colorectal cancers, and H1047L and H1047R in ovarian cancers (Fig. 2). Among Black patients with endometrial cancer, PIK3CA P539R and H1047R mutations were more and less frequent, respectively, compared to White patients (Fig. 2). Among Asian patients, PIK3CA E545K mutations were less frequent in bladder cancers (Fig. 2).

BRAF V600E mutations in Black patients were less frequent in colorectal cancers, melanoma, and thyroid cancers, but were more frequent in gliomas (Fig. 2). BRAF K601E mutations were also more frequent in colorectal cancers in Black patients. Among Asian patients, V600E mutations were less frequent in melanomas, K601E mutations were more frequent in NSCLC, and V600E mutations were more frequent in thyroid cancers (Fig. 2).

In NSCLC, EGFR exon 19 deletion, exon 20 deletion, exon 20 insertion, and T790M mutations were enriched in Black and Asian patients (Fig. 2). There were also more frequent G724S mutations and less frequent L858R mutations in Black patients, and more frequent L858R and L861Q mutations in Asian patients (Fig. 2).

### Saturation Analysis of Race-Associated Mutations

**Figure 3.**
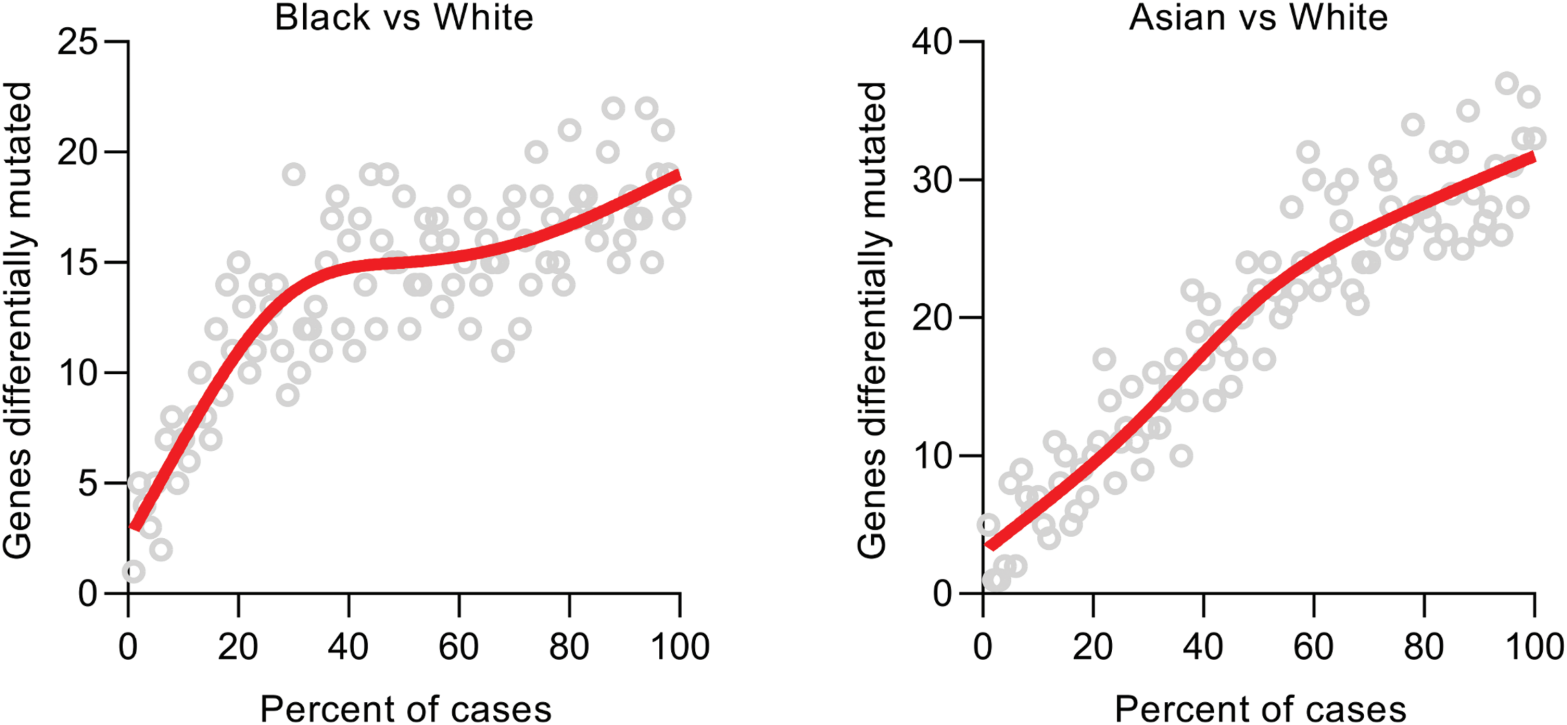
Saturation analysis of race-associated gene mutations. Down-sampling analyses were performed to quantitate discoveries in Black versus White patients and Asian versus White patients. Each point represents a random subset of patients. Red line is a smoothed fit.

To determine whether the discovery of race-associated gene-level mutations is approaching saturation, we performed a down-sampling analysis to clarify how the number of genes differentially mutated between race increases with sample size. We conducted this by repeating the pan-cancer logistic regression analysis adjusting for sex, age, and cancer type on random subsets of decreasing size in the GENIE cohort. For gene-level mutations associated with Black individuals, there was an initial rapid rise in discoveries which tapered but continues to steadily increase with sample size (Fig. 3). For gene-level mutations associated with Asian individuals, the number of discoveries increased linearly across all sample sizes (Fig. 3).

## Discussion

Leveraging the largest genomic datasets aggregated by clinical sequencing, we conducted a comprehensive analysis of genes differentially mutated between racial groups spanning multiple cancer types. This allowed us to uncover new associations and clarify previously reported associations from studies of smaller sample sizes. In particular, this study examined the largest cohort of genomically defined cancers from Black and Asian patients to date to identify tissue-specific race-associated mutations. Consequently, many race-associated mutations uncovered in this study have not been previously reported or well-investigated. Our results provide a resource to better understand the genetic contribution to disparities in cancer incidence and outcomes, and demonstrate that candidate race-associated mutations remain to be discovered.

Our study validates several established race-associated mutations including a higher prevalence of *EGFR* mutations in Asian patients with NSCLC with mutation frequencies in the GENIE cohort being similar to other large genomic studies of Asian patients (48.6% in GENIE vs 51.4% in PIONEER).^37^ We also confirmed a higher and lower frequency of *TP53* and *PIK3CA* mutations, respectively, among Black patients with breast cancer.^16,17^ Two prior pan-cancer analyses of the TCGA discovered that *TP53* and *FBXW7* mutations were more frequent in Black individuals, and our analysis confirmed the former association.^30,38^ We also substantiated that *VHL* mutations are less frequent in renal cell carcinomas of Black patients.^30^ However, we failed to replicate prior findings that *TP53* and *NFE2L2* are more frequent in esophageal cancers from Asian patients.^18^ Although the prevalence of *EGFR* mutations in Black patients in the GENIE cohort was similar to prior reports, we uncovered a greater prevalence of *EGFR* mutations in NSCLC of Black patients which contrasts studies showing no statistical difference in the frequency of NSCLC driver gene mutations between Black and White individuals.^20,29,39^ Multiple factors may account for this discrepancy including differences in the underlying demographics of patients between studies, a predominance of advanced cancers in the GENIE cohort, and the lack of data on treatment and smoking status in GENIE.

We also uncovered multiple race-associated mutations that have not been previously described but may have clinical significance. For instance, in our pan-cancer analysis of Asian versus White patients, there was an increased mutation frequency in the tumor suppressor gene *BARD1*, an interactor with BRCA1 with germline loss-of-function variants associated with familial breast cancer, and in the oncogene *FOXA1*, a transcription factor implicated in estrogen and androgen receptor signaling in treatment-resistant breast and prostate cancers.^40-44^ Asian patients were also more frequently associated with *JAK1/3* mutations, which are well characterized in blood cancers but have also been observed in solid cancers at low frequencies.^45^ The greater frequency of *TERT* promoter mutations in Black and Asian individuals in multiple cancer types may be instructive in the clinical development of drugs targeting telomerase activation as trial designs and patient recruitment strategies may only be successful if they are able to enroll and retain Asian and Black patients. Similarly, the enrichment of *FGFR3* mutations in salivary gland tumors and *ERBB4* mutations in thyroid cancers among Asian patients suggests that clinical investigations targeting these alterations need to target demographically diverse patient communities.

There are several limitations to this study. While the sample size of the GENIE dataset is larger than many other contemporary datasets, the version used in this analysis is still associated with an underrepresentation of non-White patients and is thus underpowered to detect mutational differences with more subtle effect sizes.^46,47^ In addition, this analysis was only focused on single-nucleotide variants and delins, and whether copy number alterations and structural variants may differ between patient demographics is unknown. The underlying mechanism underlying differences in mutational profiles is also unclear, and whether biological, social, or environmental/exposure-related factors may be responsible remains to be elucidated. Because our analysis could not account for genetic ancestry, future investigations are needed to assess the degree to which ancestry may account for differences in mutation profiles between patient groups, and to determine whether genetics may underly disparities in oncology treatments or outcomes.

## Supplementary Material

oyad341_suppl_Supplementary_Material

## Data Availability

The data underlying this article are available at cbioportal.org.
