# Supplementary material for: Landscape and Saturation Analysis of Mutations Associated With Race in Cancer Genomes by Clinical Sequencing
Source: Oncologist. 2024 Jan 31;29(3):219–26. doi: 10.1093/oncolo/oyad341 (PMC10911917; doi:10.1093/oncolo/oyad341)
Supplement: oyad341_suppl_Supplementary_Material [file oyad341_suppl_supplementary_material.docx]

Supplemental Table 1. Gene panel list.

| **Genes** | | | | |
| --- | --- | --- | --- | --- |
| ABL1 | CDK6 | GLI1 | MUTYH | RAD51 |
| AKT1 | CDKN1B | GNA11 | MYC | RAF1 |
| AKT2 | CDKN2A | GNAQ | MYCL | RARA |
| AKT3 | CDKN2B | GNAS | MYCN | RB1 |
| ALK | CEBPA | HNF1A | MYD88 | REL |
| APC | CHEK1 | HRAS | NBN | RET |
| AR | CHEK2 | IDH1 | NF1 | RICTOR |
| ARAF | CREBBP | IDH2 | NF2 | RNF43 |
| ARID1A | CSF1R | IGF1R | NFE2L2 | ROS1 |
| ARID1B | CTNNB1 | JAK1 | NOTCH1 | RPTOR |
| ARID2 | DDR2 | JAK2 | NOTCH2 | SDHB |
| ASXL1 | DICER1 | JAK3 | NOTCH3 | SDHD |
| ATM | DNMT3A | KDM5A | NPM1 | SETD2 |
| ATR | EGFR | KDM6A | NRAS | SF3B1 |
| ATRX | EPHA3 | KDR | NSD1 | SMAD2 |
| AURKA | ERBB2 | KEAP1 | NTRK1 | SMAD4 |
| AURKB | ERBB3 | KIT | NTRK2 | SMARCA4 |
| BAP1 | ERBB4 | KMT2A | NTRK3 | SMARCB1 |
| BARD1 | ESR1 | KMT2D | PALB2 | SMO |
| BCL2 | ETV6 | KRAS | PAX5 | SOCS1 |
| BCL6 | EZH2 | MAP2K1 | PBRM1 | SOX2 |
| BCOR | FANCA | MAP2K4 | PDGFRA | SOX9 |
| BLM | FANCC | MAP3K1 | PDGFRB | SPOP |
| BRAF | FAT1 | MCL1 | PIK3CA | SRC |
| BRCA1 | FBXW7 | MDM2 | PIK3R1 | STAG2 |
| BRCA2 | FGFR1 | MDM4 | PMS1 | STK11 |
| BRD4 | FGFR2 | MEN1 | PMS2 | SUFU |
| BRIP1 | FGFR3 | MET | POLD1 | SUZ12 |
| CARD11 | FGFR4 | MITF | POLE | TERT |
| CCND1 | FLCN | MLH1 | PRDM1 | TET1 |
| CCND2 | FLT1 | MPL | PRKAR1A | TET2 |
| CCND3 | FLT3 | MRE11 | PTCH1 | TP53 |
| CCNE1 | FLT4 | MSH2 | PTEN | TSC1 |
| CDH1 | FOXA1 | MSH6 | PTPN11 | TSC2 |
| CDK4 | GATA3 | MTOR | RAD50 |  |
| VHL | WT1 | XPO1 | U2AF1 |  |

Supplemental Figure 1. Power analysis of the GENIE cohort.


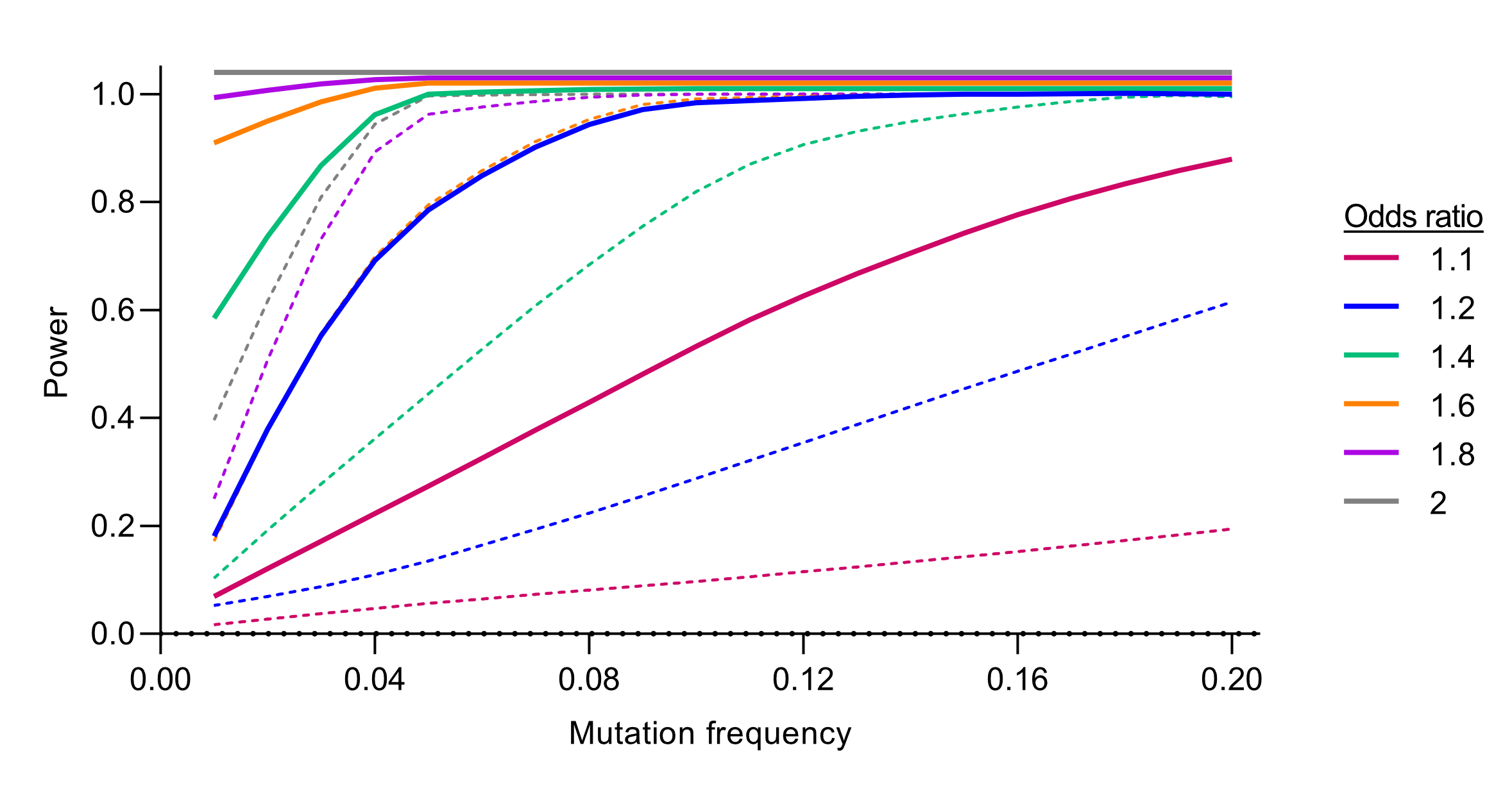


Solid lines depict estimated statistical power in a pan-cancer mutations analysis of the GENIE cohort using a type I error rate of 0.05 at various mutation frequencies and odds ratios (OR) ranging from 1.1 to 2.0. Dashed lines depict estimated statistical power in a pan-cancer mutations analysis of the TCGA cohort using a type I error rate of 0.05 at various mutation frequencies and odds ratios (OR) ranging from 1.1 to 2.0.

Supplemental Figure 2. Pan-cancer gene-level race-associated molecular alterations.


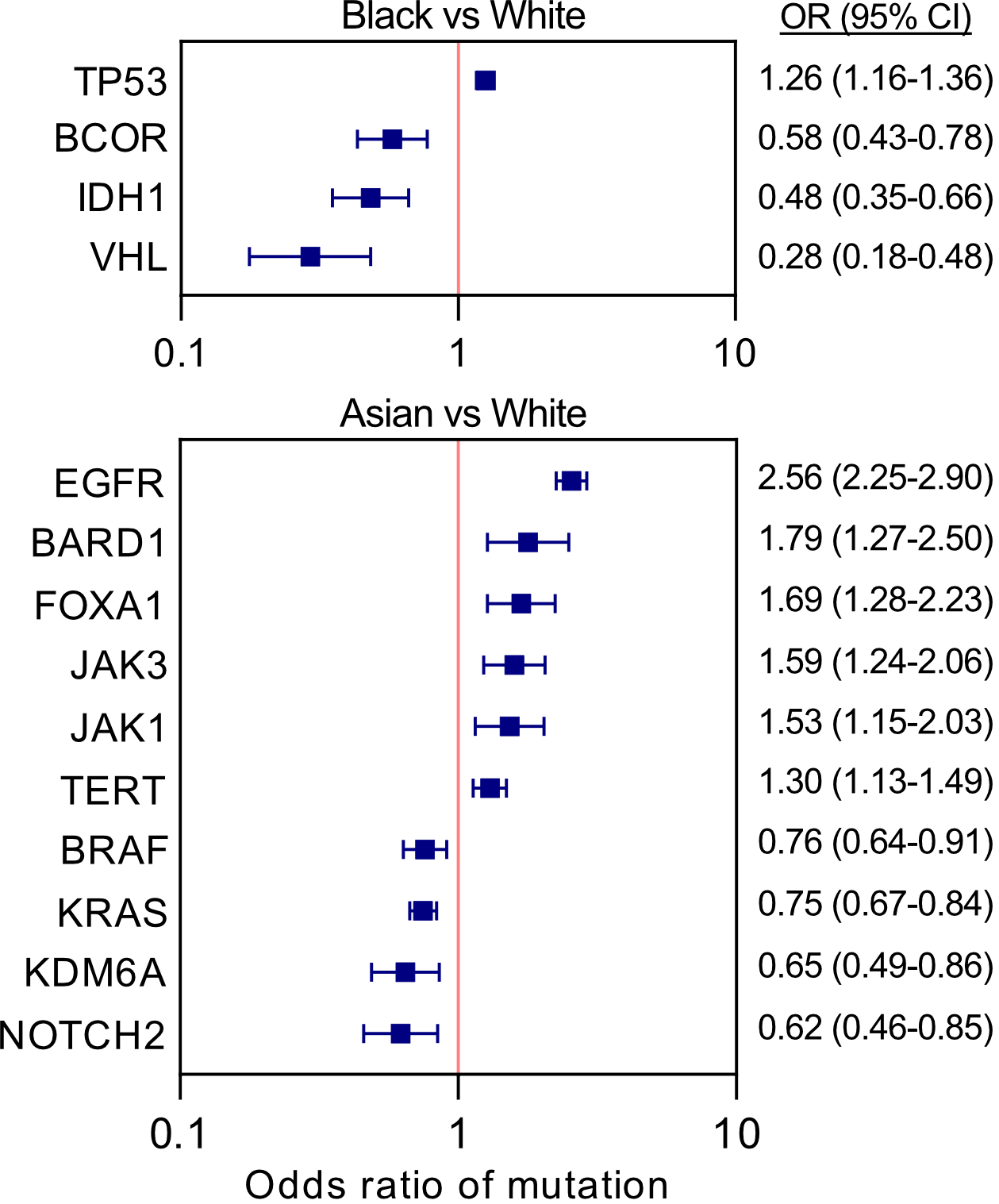


Logistic regression analysis adjusting for age, sex, cancer type, and sequencing coverage was used to determine the association between race and gene-level mutations. The significance threshold for gene associations was based on an FDR of less than 0.05.
